# ^68^Ga, ^44^Sc and ^177^Lu-labeled AAZTA^5^-PSMA-617: synthesis, radiolabeling, stability and cell binding compared to DOTA-PSMA-617 analogues

**DOI:** 10.1186/s41181-020-00107-8

**Published:** 2020-11-26

**Authors:** Jean-Philippe Sinnes, Ulrike Bauder-Wüst, Martin Schäfer, Euy Sung Moon, Klaus Kopka, Frank Rösch

**Affiliations:** 1 Johannes Gutenberg-University Mainz, Department of Chemistry/ TRIGA, Fritz-Strassmann-Weg 2, 55128 Mainz, Germany; 2 German Cancer Research Center (DKFZ), Division of Radiopharmaceutical Chemistry, Im Neuenheimer Feld 280, 69120 Heidelberg, Germany; 3 German Cancer Research Center (DKFZ), Division of Radiopharmaceutical Chemistry and German Cancer Consortium (DKTK), Im Neuenheimer Feld 280, 69120 Heidelberg, Germany; 4 New address: Helmholtz-Zentrum Dresden-Rossendorf (HZDR), Institute of Radiopharmaceutical Cancer Research, Bautzner Landstraße 400, 01328 Dresden, Germany

**Keywords:** Scandium-44, Lutetium-177, Gallium-68, AAZTA, AAZTA^5^-PSMA-617, PSMA-617, PET, Theranostics

## Abstract

**Background:**

The AAZTA chelator and in particular its bifunctional derivative AAZTA^5^ was recently investigated to demonstrate unique capabilities to complex diagnostic and therapeutic trivalent radiometals under mild conditions. This study presents a comparison of ^68^Ga, ^44^Sc and ^177^Lu-labeled AAZTA^5^-PSMA-617 with DOTA-PSMA-617 analogues. We evaluated the radiolabeling characteristics, in vitro stability of the radiolabeled compounds and evaluated their binding affinity and internalization behavior on LNCaP tumor cells in direct comparison to the radiolabeled DOTA-conjugated PSMA-617 analogs.

**Results:**

AAZTA^5^ was synthesized in a five-step synthesis and coupled to the PSMA-617 backbone on solid phase. Radiochemical evaluation of AAZTA^5^-PSMA-617 with ^68^Ga, ^44^Sc and ^177^Lu achieved quantitative radiolabeling of > 99% after less than 5 min at room temperature. Stabilities against human serum, PBS buffer and EDTA and DTPA solutions were analyzed. While there was a small degradation of the ^68^Ga complex over 2 h in human serum, PBS and EDTA/DTPA, the ^44^Sc and ^177^Lu complexes were stable at 2 h and remained stable over 8 h and 1 day. For all three compounds, i.e. [^nat^Ga]Ga-AAZTA^5^-PSMA-617, [^nat^Sc]Sc-AAZTA^5^-PSMA-617 and [^nat^Lu]Lu-AAZTA^5^-PSMA-617, in vitro studies on PSMA-positive LNCaP cells were performed in direct comparison to radiolabeled DOTA-PSMA-617 yielding the corresponding inhibition constants (K_i_). K_i_ values were in the range of 8–31 nM values which correspond with those of [^nat^Ga]Ga-DOTA-PSMA-617, [^nat^Sc]Sc-DOTA-PSMA-617 and [^nat^Lu]Lu-DOTA-PSMA-617, i.e. 5–7 nM, respectively. Internalization studies demonstrated cellular membrane to internalization ratios for the radiolabeled ^68^Ga, ^44^Sc and ^177^Lu-AAZTA5-PSMA-617 tracers (13–20%IA/10^6^ cells) in the same range as the ones of the three radiolabeled DOTA-PSMA-617 tracers (17–20%IA/10^6^ cells) in the same assay.

**Conclusions:**

The AAZTA^5^-PSMA-617 structure proved fast and quantitative radiolabeling with all three radiometal complexes at room temperature, excellent stability with ^44^Sc, very high stability with ^177^Lu and medium stability with ^68^Ga in human serum, PBS and EDTA/DTPA solutions. All three AAZTA^5^-PSMA-617 tracers showed binding affinities and internalization ratios in LNCaP cells comparable with that of radiolabeled DOTA-PSMA-617 analogues. Therefore, the exchange of the chelator DOTA with AAZTA^5^ within the PSMA-617 binding motif has no negative influence on in vitro LNCaP cell binding characteristics. In combination with the faster and milder radiolabeling features, AAZTA^5^-PSMA-617 thus demonstrates promising potential for in vivo application for theranostics of prostate cancer.

## Background

The new era of radio-theranostics in (nuclear) medicine is driven in a significant way by potent radiolabeled PSMA (prostate-specific membrane antigen) inhibitors based on the KuE (lysine-urea-glutamate) motif binding to prostate cancer cells. This success was initiated by [^68^Ga]Ga-HBED-PSMA ([^68^Ga]Ga-PSMA-11) for PET/CT imaging, while the important therapeutic compounds are DOTA (1,4,7,10-Tetraazacyclododecane-1,4,7,10-tetraacetic acid)-conjugated KuE derivatives with varying linker and spacer moieties, ready for labelling with ^90^Y, ^177^Lu and other radiolanthanides, as well as alpha emitters such as ^225^Ac. One of the relevant features of the diagnostic tracer [^68^Ga]Ga-HBED-PSMA is its easy labeling under mild conditions (Eder et al. 2012a), which later was paralleled by DATA (2,2′-(6-((carboxymethyl)amino)-1,4-diazepane-1,4-diyl) diacetic acid)- and THP-conjugated KuE motifs (Tsionou et al. 2017; Seemann et al. 2015a; Nagy et al. 2017; Pfister et al. 2015). In contrast, the various therapeutic analogs all come with DOTA as chelator, which requires radiolabeling at about 95 °C (Benešová et al. 2015).

The rational of the present study was to substitute the macrocycle DOTA by the hybrid chelator AAZTA (1,4-Bis (carboxymethyl)-6-[bis (carboxymethyl)]amino-6-methylperhydro-1,4-diazepine), known for its possibility to complex trivalent (radio) metals under mild temperatures, such as ^68^Ga (*β*^+^ = 89%, t_1/2_: 68 min), ^44^Sc (*β*^+^ = 94%, t_1/2_: 4.0 h) and ^177^Lu (*β*^−^ = 100%, t_1/2_: 6.6 d). The bifunctional chelator AAZTA^5^ has been developed in direct comparison to DATA^5m^ (Seemann et al. 2017). AAZTA^5^-TOC was recently described and evaluated in terms of radiolabeling capabilities and stabilities of the complexes with ^68^Ga, ^44^Sc and ^177^Lu (Sinnes et al. 2019).

The two basic questions addressed in the present study are 1. what are the radiolabeling and in vitro stability characteristics for [^68^Ga]Ga-AAZTA^5^-PSMA-617, [^44^Sc]Sc-AAZTA^5^-PSMA-617 and [^177^Lu]Lu-AAZTA^5^-PSMA-617, and 2. how may the exchange of AAZTA for DOTA in the same targeting vector PSMA-617 influence the in vitro binding of the new compounds to prostate cancer cells in vitro.

Concerning complex formation aspects, there is a strong difference in complex geometry and amount of donor sites the chelator has to offer for stable complexation especially between ^68^Ga and ^44^Sc, two metals from the fourth period, and ^177^Lu, as part of the lanthanides in the fifth period. Whereas gallium complexes in general need 6 coordination sites, scandium requires at least 7 coordination and lutetium prefer a coordination with up to 8 donor atoms (Nagy et al. 2017; Parker et al. 2013; Aime et al. 1996), so one might expect different answers for question 1.

Concerning the possible impact of the chelator on the radiopharmacology of the same targeting vector, there have been a number of systematic studies for various targeting vectors indicating, that the “chelator makes a difference” (Fani et al. 2011, 2012).

**Fig. 1:**
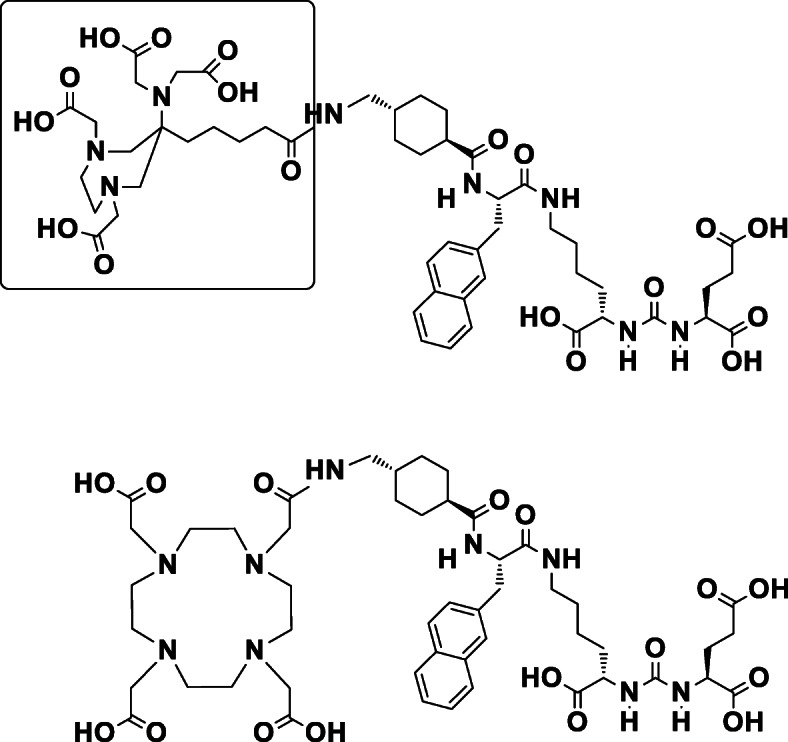
AAZTA^5^-PSMA-617 and DOTA-PSMA-617, AAZTA^5^-chelator highlighted

In the case of AAZTA, we recently reported promising results for radiolabeled AAZTA^5^-TOC directly compared with DOTA-TOC (Sinnes et al. 2019). This encouraged us to investigate whether the exchange of DOTA to AAZTA^5^ conjugated to the Glu-urea-Lys binding motif through the 2-naphthyl-L-Ala-AMCH linker of PSMA-617, cf. Fig. 1, may affect the cell binding of the targeting vector or not. For PSMA derivatives, a well evaluated in vitro affinity assay based on LNCaP cells has been recently developed (Benešová et al. 2015). Data have been published already for ^68^Ga-, ^44^Sc- and ^177^Lu-labeled DOTA-PSMA-617 (Benešová et al. 2015, 2016; Umbricht et al. 2017), which can be used for direct comparison to the new AAZTA^5^-PSMA-617 derivatives.

## Materials and methods

### Synthesis of AAZTA^5^-PSMA (AAZTA^5^-Chx-2-Nal-Lys-Urea-Glu)

**Fig. 2:**
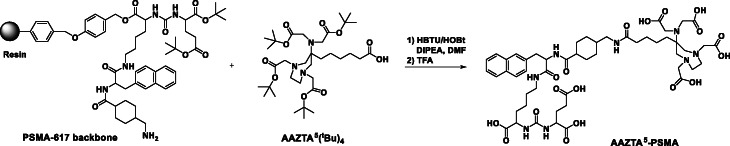
Reaction of AAZTA^5^(^t^Bu_4_) with PSMA-617 on resin via amide coupling forming AAZTA^5^-PSMA. AAZTA^5^(^t^Bu_4_) was synthesized according (Sinnes et al. 2019) and PSMA-617 following (Benešová et al. 2016)

AAZTA^5^(^t^Bu)_4_ was synthesized in five steps according to the protocol from Sinnes et al. yielding a ready-for-coupling derivative (Sinnes et al. 2019). The PSMA-617 backbone was synthesized on solid phase following the established procedure from Heidelberg (Benešová et al. 2016). A standard amide coupling using HBTU/HOBt and DIPEA was performed to couple the AAZTA^5^ to PSMA on solid phase. AAZTA^5^(^t^Bu)_4_ (59.0 mg; 0.09 mmol) was mixed with HATU (33.4 mg; 0.09 mmol), HOBt (35.6 mg; 0.26 mmol) and DIPEA (45 μl; 0.26 mmol) in dry DMF (2 mL) and shaken for 20 min. The solution was added to PSMA-617 (resin) (65.0 mg; 0.06 mmol) soaked in dry DMF (1 mL) and shaken overnight at RT (Fig. 2). After completion of the reaction, the solution was filtered off and the solid was washed with DCM. TFA (3 × 2 mL) was added to the resin and shaken for 20 min. The TFA solutions were combined and stirred for 4 h at room temperature until completely deprotection. The solution was concentrated under vacuum and after HPLC (High-performance liquid chromatography) purification [LUNA column (Phenomenex® Luna® 10 μm C18(2) 100 Å) with a slow gradient of 25–30% MeCN (+ 0.1% TFA) / 75–70% water (+ 0.1% TFA), *t*_*R*_ = 16.8 min.], a white solid was obtained (6.6 mg; 0.006 mmol; 12%) MS (ESI^+^) : 1085:5 (M + H^+^); calculated for C_51_H_72_N_8_O_18_ : 1084:50.

### Radiolabeling with ^68^Ga, ^44^Sc and ^177^Lu

For radiochemical evaluation with ^68^Ga, a ^68^Ge/^68^Ga generator (TiO_2_-based matrix, Cyclotron Co. Obninsk, Russia) was used with online acetone post-processing separating iron and zinc impurities as well as ^68^Ge breakthrough (Zhernosekov et al. 2007; Seemann et al. 2015b). Gallium-68 radiolabeling for in vitro studies was performed with a ^68^Ge/^68^Ga generator based on a pyrogallol resin (Schuhmacher and Maier-Borst 1981). Radiolabeling with ^44^Sc was performed with a 150 MBq ^44^Sc/^44^Ti generator (Filosofov et al. 2010; Pruszyński et al. 2010). ^177^Lu, produced via the ^176^Yb-pathway (Lebedev et al. 2000), was provided by ITG Munich.

Labeling with ^68^Ga and ^177^Lu were performed in 1 ml of 0.2 M ammonium acetate buffer pH 4.5 at room temperature (25 °C). For ^44^Sc, due to the general post processing, the radionuclide is provided in 0.25 M ammonium acetate buffer pH 4 (Pruszyński et al. 2010). Kinetic studies were done with 50 MBq for ^44^Sc and 100 MBq for ^68^Ga and ^177^Lu and aliquot were taken at different time points 1, 3, 5 and 10 min. The pH was controlled at start of labeling and after labeling was finished.

For reaction control thin-layer chromatography (TLC) with citrate buffer, pH 7, as eluent and radio HPLC (Merck Chromolith® RP-18e-column, water: MeCN with 0.1% TFA, 5 to 95% MeCN in 10 min) was used. TLC’s were measured in RITA TLC imager (Elysia Raytest). The citrate TLC showed free radiometal with a R_f_ of 0.9 and all labeled compounds were located at R_f_ of 0.1 to 0.3. Radio-HPLC was used to characterize the labeled conjugates and to exclude the presence of colloidal radiometals not visible on TLC.

### Stability studies

Stability studies were performed in human serum (HS), phosphate buffered saline (PBS) and EDTA/DTPA solution (pH adjusted to 7 by PBS buffer) in triplicate. Only batches obtained with > 95% labeling yield were used and time points were adjusted to the radionuclides’ physical half-life: ^68^Ga – 0.5, 1, 2 h; ^44^Sc – 0.5, 1, 4, 8, 24 h; ^177^Lu − 1, 2, 4, 24 h. HS (human male AB plasma, USA origin) were bought from Sigma Aldrich, PBS was prepared with a BupH™ Phosphate Buffered Saline Pack (PIERCE), EDTA/DTPA solution were prepared using the prepared PBS buffer and adding ETDA/DTPA to a 0.01 M concentration. Final procedure used 50–70 μl of the labeling solution added to 1 ml of stability solution. Final pH was controlled to ensure no influence of the labeling buffer on the stability solution.

### Affinity studies/ cell binding studies

The in vitro experiments were performed using the PSMA-positive LNCaP cell line (androgen-sensitive human lymph node metastatic lesion of prostatic adenocarcinoma, CRL-1740 [American Type Culture Collection]) (Benešová et al. 2015). For negative control, PSMA-PC3 cells (bone metastasis of a grade IV prostatic adenocarcinoma, ATCC CRL-1435) were used (Eder et al. 2012a). The cells were cultured in RPMI1640 medium supplemented with 10% fetal calf serum and L-glutamine and incubated at 37 °C in an environment of humidified air containing 5% CO_2_. The cells were harvested using trypsin-ethylenediaminetetraacetic acid (trypsin-EDTA; 0.05% trypsin, 0.02% EDTA, all from PAN Biotech).

Cell binding affinity was determined by competitive cell binding assay (Eder et al. 2012a). 10^5^ LNCaP cells per well were incubated with 0.75 nM [^68^Ga]Ga-PSMA-10 in presence of 12 different concentrations (0–5000 nM) of cold complexes (natural Ga, Sc and Lu) of AAZTA^5^-PSMA-617 in a volume of 100 μL by shaking for 45 min at room temperature and then removed using a multiscreen vacuum manifold (Millipore, Billerica, MA). Afterwards the cells were washed twice with 100 μL and once with 200 μL binding buffer at 5 °C. The cell containing filters were stamped out and measured in a gamma counter (Packard Cobra II, GMI, Minnesota, USA). Using a nonlinear regression algorithm (GraphPad Prism Software) the 50% inhibitory concentration (IC_50_) values were calculated. Each sample was done in quadruple while the whole experiment was done three times.

Nonradioactive complexes of the AAZTA^5^-PSMA-617 with ^nat^Ga, ^nat^Sc and ^nat^Lu were synthesized by adding 15 μL (150 nmol) of a 10 mM solution of the metal chlorides to 100 nmol of chelator in 85 μL (1.5: 1 = metal: chelator) and filling up to 200 μL with 0.2 M NH_4_Ac buffer pH 4.5. The solution of 0.2 M NH_4_Ac buffer pH 4.5 with a final concentration of 500 μM AAZTA^5^-PSMA-617 was shaken for 20 min at room temperature (25 °C) and quantitative complexation was reached after 20 min monitored by ESI LC-MS. MS (ESI^+^) for [^nat^GA]Ga−AAZTA^5^−PSMA: 1151.3 [M+H]^+^, 576.3 [M+2H]^2+^; calculated for C_51_H_69_GaN_8_O_18_: 1150.4; MS (ESI^+^) for [^nat^Sc]Sc−AAZTA^5^−PSMA: 1127.4 [M+H]^+^, 564.3 [M+2H]^2+^; calculated for C_51_H_68_ScN_8_O_18_: 1126.1; MS (ESI^+^) for [^nat^Lu]Lu−AAZTA^5^−PSMA: 1257.6 [M+H]^+^, 629.3 [M+2H]^2+^; calculated for C_51_H_68_LuN_8_O_18_: 1256.1

All AAZTA^5^-PSMA-617 complexes were tested 3 times in triplicate leading to *n* = 9. Same was done with the PSMA-617 complexes on the same cell-plates.

### Internalization studies

For internalization studies 10^5^ LNCaP or PC3 cells were seeded in poly(L-lysine)-coated 24-well cell culture plates at 37 °C in an environment of humidified air containing 5% CO_2_ for 24 h. (Mier et al. 2007). The medium is removed and 250 μl of radiolabeled 30 nM AAZTA^5^-PSMA is replaced for 45 min. One plate is being incubated at 37 °C and the second one at 4 °C to inhibit the internalization. The specificity of the ligands is proofed by addition 500 μM of 2-(phosphonomethyl)-pentanedioic acid (2-PMPA, Axxora, Loerrach, Germany). After incubation, the cells were washed three times with 1 ml ice cold PBS. To determine the surface-bound activity cells were incubated twice with 0.5 ml of glycine-HCl in PBS (50 mM, pH 2.8) each for 5 min at room temperature. Both washing steps were collected for measuring with a gamma counter (Packard Cobra II, GMI, Minnesota, USA). Before lysating the cells with 0.5 mL of 0.3 M NaOH to determine the internalized fraction, they were once washed with 1 ml ice cold PBS (Eder et al. 2012a).

## Results

### Radiolabeling and stability studies

AAZTA^5^-PSMA-617 (AAZTA^5^-Chx-2-NaI-Lys-Urea-Glu) was successfully radiolabeled in quantitative radiochemical yields (> 99%, as determined both by radio-HPLC and radio-TLC) with ^68^Ga, ^44^Sc and ^177^Lu, in less than 5 min at room temperature. Precursor amounts were optimized to 5 nmol (5.4 μg) for ^68^Ga and ^44^Sc and 0.6 nmol (0.65 μg) for ^177^Lu (molar ratio 10: 1 for chelator to radiometal). Final concentrations for the internalization studies of 6 μM (6 nmol/ml) were easily reached and final radiochemical purities of 99.9% (as determined by radio-HPLC) allowing direct use of the obtained product solution for the in vitro assay without further purification.

The stability studies, against HS, PBS buffer and EDTA/DTPA in PBS buffer, showed [^68^Ga]Ga-AAZTA^5^-PSMA-617 to be > 95% stable against HS and > 90% stable in PBS and EDTA/DTPA in PBS over 2 h. [^44^Sc]Sc-AAZTA^5^-PSMA-617 was completely stable with > 95% over 8 h against HS, PBS and EDTA/DTPA in PBS and even stayed stable with > 95% after 24 h against HS. The ^177^Lu complex stabilities at 2 h were > 98%, while small degradations to 81% were observed after 24 h against HS. Stabilities of [^177^Lu]Lu-AAZTA^5^-PSMA-617 against PBS and EDTA/DTPA in PBS are between 85 and 90% after 24 h.

### In vitro binding affinity and internalization studies

In competitive binding studies against [^68^Ga]Ga-PSMA-10, all three non-radioactive metal complexes of AAZTA^5^-PSMA-617 indicated nanomolar binding affinities: 8.7 ± 0.8 nM for the gallium complex, 30.6 ± 11.5 nM for the scandium complex and 26.6 ± 11.1 nM for the lutetium complex. By applying the Cheng-Prusov equation on the IC_50_ values the following inhibition constants were obtained (Table 1) (Craig 1993).

**Table 1:** Inhibition constants (K_i_ in nM) and internalization values (%IA = % internalized activity) of [^nat/68^Ga]Ga-PSMA-11, [^nat/68^Ga]Ga-, [^nat/44^Sc]Sc-, [^nat/177^Lu]Lu-DOTA-PSMA-617 and [^nat/68^Ga]Ga-, [^nat/44^Sc]Sc-, [^nat/177^Lu]Lu-AAZTA^5^-PSMA-617

derivative	K_i_ ± SD / nM		Internalization ± SD / %IA/10^6^ cells	
[^nat/68^Ga]Ga-PSMA-11	12.0 ± 2.8	(Eder et al. 2012a)	9.5 ± 2.6	(Benešová et al. 2015)
[^nat/68^Ga]Ga-PSMA-617	6.4 ± 1.0	(Benešová et al. 2015)	17.7 ± 4.4	(Benešová et al. 2015)
[^nat/68^Ga]Ga-AAZTA^5^-PSMA	8.7 ± 0.9		13.0 ± 0.2	
[^nat/44^Sc]Sc-PSMA-617	4.7 ± 0.8	(Eppard et al. 2017)	15.8 ± 2.1	(Eppard et al. 2017)
[^nat/44^Sc]Sc-AAZTA^5^-PSMA	30.6 ± 11.5		20.0 ± 0.9	
[^nat/177^Lu]Lu-PSMA-617	6.9 ± 1.3	(Benešová et al. 2015)	17.5 ± 3.1	(Benešová et al. 2015)
[^nat/177^Lu]Lu-AAZTA^5^-PSMA	26.6 ± 11.1		18.0 ± 2.0	

$$ {\mathrm{K}}_{\mathrm{i}}=\frac{\mathrm{IC}50}{\left(1+\frac{{\mathrm{C}}_{\mathrm{d}\mathrm{im}}}{{\mathrm{K}}_{\mathrm{d}}}\right)} $$

All three radiolabeled AAZTA^5^-PSMA-617 derivatives internalized well with 13–20%IA / 10^6^ cells (Table 1 / Fig. 1), which is comparable to the internalization of the DOTA-PSMA-617 analogs published elsewhere (Benešová et al. 2015; Eppard et al. 2017). For all compounds the blocking with 2-PMPA was successful at 37 °C. All AAZTA^5^-PSMA-617 complexes showed high cell surface binding on 37 °C comparable to the PSMA-617 complexes (37 °C and 4 °C values shown in supplements, Figs. 1 and 2). Comparing surface to internalized activity lead to %internalization / total cellular activity ratios of 43% for [^68^Ga]Ga-AAZTA^5^-PSMA-617 comparable to the 47% value reported for [^68^Ga]Ga-DOTA-PSMA-617 (Benešová et al. 2015). The %internalization / total cellular activity ratio for the [^44^Sc]Sc-AAZTA^5^-PSMA-617 with 41% is in the same range as the ^68^Ga-complex. The value is lower compared to the measured value of 51% for [^44^Sc]Sc-PSMA-617 in the same assay, but very similar with the literature value of 41% published by Eppard et al. (Eppard et al. 2017). For [^177^Lu]Lu-AAZTA^5^-PSMA-617 the %internalization / total celluar activity ratio of 36% was higher than the measured 27% value for [^177^Lu]Lu-PSMA-617 in the same assay, but much higher than the published value of 10–15% for [^177^Lu]Lu-PSMA-617 from Umbricht et al. (Umbricht et al. 2017).

**Fig. 3:**
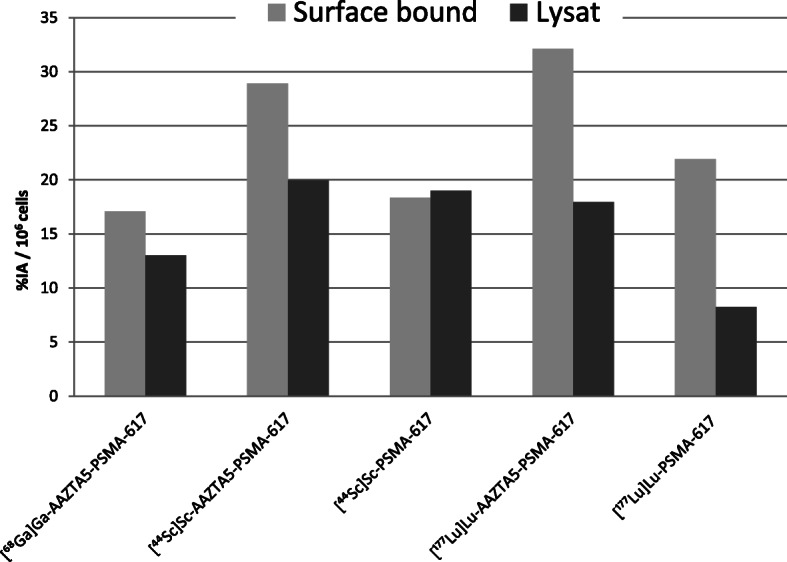
Internalization data of the ^68^Ga, ^44^Sc and ^177^Lu complexes of AAZTA^5^-PSMA-617 and PSMA-617 in LNCaP cells at 37 °C Cheng-Prusov equation with: c_dim_ = concentration of PSMA-10 and K_d_ = dissociation constant of [^68^Ga]Ga-PSMA-10

Experiments on negative PC3 cells proved no specific internalization or cell surface binding for both [^44^Sc]Sc-AAZTA^5^-PSMA-617 and [^177^Lu]Lu-AAZTA^5^-PSMA-617. Also, the ^44^Sc and ^177^Lu complexes of the free chelator AAZTA^5^ were tested on LNCaP-cells showing no specific internalization or cell surface binding (data shown in Supplements) (Fig. 3).

## Discussion

### Radiochemistry

Quantitative radiolabeling with ^68^Ga, ^44^Sc and ^177^Lu was achieved at room temperature for AAZTA^5^-PSMA-617 within less than 5 min at room temperature. This corresponds with literature data for the free chelator (Nagy et al. 2017) as well as with recent evaluations of AAZTA^5^-TOC (Sinnes et al. 2019).

### Stability studies

[^68^Ga]Ga-AAZTA^5^-PSMA-617 is stable in vitro against HS with very small degradation over 2 h against PBS, cf. Table 1 in the supplement. This corresponds to values of [^68^Ga]Ga-AAZTA^5^-TOC (Sinnes et al. 2019) where conjugating the targeting molecule TOC to the chelator even stabilized the complex. The low-molecular weight molecule PSMA-617 seems to have the same stabilizing influence on the chelator as the cyclic peptide TOC, but to a smaller extend (Table 2). [^68^Ga]Ga-AAZTA^5^-PSMA-617 was not as stable as [^68^Ga]Ga-AAZTA^5^-TOC, but still more stable compared to the ^68^Ga complex with the free chelator, i.e. [^68^Ga]Ga-AAZTA^5^.

**Table 2:** Stability values of ^68^Ga complexes of AAZTA^5^, AAZTA^5^-TOC and AAZTA^5^-PSMA-617

	time	[^68^Ga]Ga- AAZTA^5^	[^68^Ga]Ga-AAZTA^5^-TOC	[^68^Ga]Ga- AAZTA^5^-PSMA-617
HS	0.5 h	93.0 ± 0.8	98.1 ± 0.2	94.7 ± 0.3
	1 h	92.7 ± 1.3	96.9 ± 0.2	94.3 ± 0.4
	2 h	85.3 ± 3.4	95.0 ± 0.3	91.3 ± 0.7
PBS	0.5 h	97.5 ± 1.0	99.1 ± 0.2	94.1 ± 0.2
	1 h	88.7 ± 0.5	99.2 ± 0.1	92.7 ± 0.5
	2 h	78.5 ± 4.5	99.3 ± 0.1	92.8 ± 0.6

[^44^Sc]Sc-AAZTA^5^-PSMA-617 was completely stable over 8 h with no degradation in HS even after 24 h. This corresponds with the literature showing a high stability of the Sc-AAZTA complex (Nagy et al. 2017; Sinnes et al. 2019). The high stability in HS, PBS and EDTA/DTPA in PBS is one of the prerequisites that justifies first preclinical in vivo applications in the next steps (in Supplement Table 2, also with EDTA/DTPA-values). [^177^Lu]Lu-AAZTA^5^-PSMA-617 displays high stability over 8 h, yet starts with degradation slowly after 24 h.

### Binding affinity

The binding affinities for all three non-radioactive metal complexes of AAZTA^5^-PSMA-617 are in the low nM range. [^nat^Ga]Ga-AAZTA^5^-PSMA-617 reached the same K_i_ value range of 8–9 nM as the PSMA-617 analogue, while proving a higher binding affinity as [^nat^Ga]Ga-PSMA-11.

Both [^nat^Sc]Sc-AAZTA^5^-PSMA-617 and [^nat^Lu]Lu-AAZTA^5^-PSMA-617 displayed a somewhat lower affinity of 31 and 27 nM, respectively. The most noticeable difference, beside AAZTA^5^ being a hybrid chelator, i.e. shows both cyclic and acyclic features, what mostly effects the radiochemistry, is the charge of − 1 for the AAZTA^5^ scandium and lutetium complexes. For DOTA, the necessary donor set consists of N_4_O_2_ to N_4_O_4_, and at least one of the acid groups is part of the bifunctionalization to the targeting molecule. As a M^3+^ radiometal DOTA is forming a neutral complex, whereas AAZTA^5^ with a N_3_O_4_ core forms an overall negative charged complex (net charge − 1). This negative charge is created by the 4 acid groups that need to be deprotected for complexation and binding to the radiometal. The four negative charges of the acid groups overcompensate for the three positive charges of the radiometal. The complex of the gallium can be neutral because gallium may not require all donor atoms from the AAZTA^5^ since gallium prefers an octahedral coordination sphere leaving one acid group empty. The negative charge definitely may have an influence on the in vitro as well as the in vivo behavior of the targeting molecule. Besides however, both chelator systems provide a suitable scaffold for a more efficient locally arrangement of the donor atoms (nitrogen and oxygens atoms) to achieve metal coordination. A relevant influence on the host of the radiometal inside the chelator ring arises the entropic effect.

The relatively high SD for both affinity and internalization in case of the scandium and the lutetium complexes are justified by 9 total measurements over 9 experimental days (SD on each day ±1–2), whereas the gallium complex was tested twice on the same day. With each measurement being quadruple, total repetition was *n* = 8 for the gallium complex and *n* = 36 for the scandium and the lutetium complex, respectively.

### Internalization

All AAZTA^5^-PSMA complexes internalize well and this was demonstrated to be a specific and active process. The internalization value for [^68^Ga]Ga-AAZTA^5^-PSMA-617 is in the same range as literature values of [^68^Ga]Ga-PSMA-617, with both reaching internalization ratios around 45%. The [^44^Sc]Sc-AAZTA^5^-PSMA reached ratios of 41% internalized activity while [^44^Sc]Sc-PSMA-617 showed up to 50% on the same day, while literature also giving around 41% internalized activity. For [^177^Lu]Lu-AAZTA^5^-PSMA-617 ratios of 36% internalized activity were measured, which are higher than the 27% measured for [^177^Lu]Lu-PSMA-617. Literature values for [^177^Lu]Lu-PSMA-617 of 10–15% are much lower than the measured values in this assay.

Surface binding obtained for the radiolabeled AAZTA^5^-PSMA-617 complexes could be blocked by 2-PMPA. Similarly, [^44^Sc]Sc-AAZTA^5^ and [^177^Lu]Lu-AAZTA^5^ showed no surface activity or internalization on PSMA positive LNCaP cells and on PC3 cells as a second negative experiment.

## Conclusion

Radiolabeled AAZTA^5^-PSMA-617 conjugates guarantee for almost quantitative yields of > 99% of ^44^Sc-, ^68^Ga- and ^177^Lu-labeling under mild conditions in short time after less than 5 min at room temperature. Subsequent purification is obsolete for in vitro studies. [^68^Ga]Ga-AAZTA^5^-PSMA-617, [^44^Sc]Sc-AAZTA^5^-PSMA-617 and [^177^Lu]Lu-AAZTA^5^-PSMA-617 are stable to a different degree, with favorable in vitro stabilities in particular for the ^44^Sc and ^177^Lu versions.

These synthetic advantages in terms of radiolabeling and stability are accompanied by good binding affinities in vitro and excellent PSMA-specific internalization in LNCaP tumor cells, which correspond with those of the radiolabeled PSMA-617 versions. Therefore, the exchange of the chelator DOTA with AAZTA^5^ within the chelator-PSMA-617 binding motif has no negative influence on in vitro LNCaP cell binding characteristics. In combination with the faster and milder radiolabeling features, AAZTA^5^-PSMA-617 thus demonstrates potential for in vivo application for theranostics of prostate cancer, in particular for [^44^Sc]Sc-AAZTA^5^-PSMA-617 and [^177^Lu]Lu-AAZTA^5^-PSMA-617.

## Additional file

**Additional file 1: Figure 1.** Internalization data of the ^44^Sc complexes of AAZTA^5^-PSMA-617, AAZTA^5^ and PSMA-617 in PC3 cells at 37 °C and 4 °C. **Figure 2**. Internalization data of the ^177^Lu complexes of AAZTA^5^-PSMA-617, AAZTA^5^ and PSMA-617 in PC3 cells at 37 °C and 4 °C. **Figure 3.** Internalization data of the ^68^Ga, ^44^Sc and ^177^Lu complexes of AAZTA^5^-PSMA-617 and PSMA-617 in LNCaP cells at 37 °C and 4 °C. **Figure 4.** radio-HPLC spectra of [^68^Ga]Ga-AAZTA^5^-PSMA with linear gradient condition of 5–95% MeCN (+ 0.1% TFA)/95–5% Water (+ 0.1% TFA) in 10 min, 1 mL/min, t_R_ = 10 min. **Figure 5**. radio-HPLC spectra of [^44^Sc]Sc-AAZTA^5^-PSMA with linear gradient condition of 5–95% MeCN (+ 0.1% TFA)/95–5% Water (+ 0.1% TFA) in 10 min, 1 mL/min, t_R_ = 10.5 min. **Figure 6**. radio-HPLC spectra of [^177^Lu]Lu-AAZTA^5^-PSMA with linear gradient condition of 5–95% MeCN (+ 0.1% TFA)/95–5% Water (+ 0.1% TFA) in 10 min, 1 mL/min, t_R_ = 10 min. **Table 1**. Stability values of ^68^Ga complexes of AAZTA^5^, AAZTA^5^-TOC and AAZTA^5^-PSMA-617. **Table 2**. Stability values of ^44^Sc and ^177^Lu complexes of AAZTA^5^, AAZTA^5^-TOC and AAZTA^5^-PSMA-617

## Data Availability

The datasets generated and/or analyzed during the current study are available from the corresponding author on reasonable request.

## Abbreviations

AAZTA: 1,4-Bis (carboxymethyl)-6-[bis (carboxymethyl)]amino-6-methylperhydro-1,4-diazepine
AAZTA^5^: 1,4-Bis (carboxymethyl)-6-[bis (carboxymethyl)]amino-6-[pentanoic-acid]perhydro-1,4-diazepine
DTPA: Diethylenetriaminepentaacetic acid
DIPEA: *N*,*N*-Diisopropylethylamine
DOTA: 1,4,7,10-Tetraazacyclododecane-1,4,7,10-tetraacetic acid
HBTU: 2-(1*H*-Benzotriazol-1-yl)-1,1,3,3-tetramethyluronium hexafluorophosphate
HOBt: Hydroxybenzotriazole
HPLC: High-performance liquid chromatography
LC: Liquid chromatography
MS: Mass spectrometry
n.c.a.: No-carrier-added
NH_4_OAc: Ammonium acetate
HS: Human serum
PBS: Phosphate buffered saline
RCY: Radiochemical yield
RT: Room temperature
TLC: Thin layer chromatography
DATA: 2,2’-(6-((carboxymethyl)amino)-1,4-diazepane-1,4-diyl) diacetic acid
EDTA: Ethylenediaminetetraacetic acid
IC_50_: Half-maximal inhibitory concentration
PSMA: Prostate-specific membrane antigen
K_i_: Inhibition constant
%IA: Internalized activity
HBED: *N,N′*-Bis (2-hydroxybenzyl)ethylenediamine-*N,N′*-diacetic acid
KuE: Lysine-urea-glutamate
