# Supplementary material for: 68Ga, 44Sc and 177Lu-labeled AAZTA5-PSMA-617: synthesis, radiolabeling, stability and cell binding compared to DOTA-PSMA-617 analogues
Source: EJNMMI Radiopharm Chem. 2020 Nov 26;5:28. doi: 10.1186/s41181-020-00107-8 (PMC7691401; doi:10.1186/s41181-020-00107-8)
Supplement: Supplementary file 1 — Additional file 1: Figure 1. Internalization data of the 44Sc complexes of AAZTA5-PSMA-617, AAZTA5 and PSMA-617 in PC3 cells at 37 °C and 4 °C. Figure 2. Internalization data of the 177Lu complexes of AAZTA5-PSMA-617, AAZTA5 and PSMA-617 in PC3 cells at 37 °C and 4 °C. Figure 3. Internalization data of the 68Ga, 44Sc and 177Lu complexes of AAZTA5-PSMA-617 and PSMA-617 in LNCaP cells at 37 °C and 4 °C. Figure 4. radio-HPLC spectra of [68Ga]Ga-AAZTA5-PSMA with linear gradient condition of 5–95% MeCN (+ 0.1% TFA)/95–5% Water (+ 0.1% TFA) in 10 min, 1 mL/min, tR = 10 min. Figure 5. radio-HPLC spectra of [44Sc]Sc-AAZTA5-PSMA with linear gradient condition of 5–95% MeCN (+ 0.1% TFA)/95–5% Water (+ 0.1% TFA) in 10 min, 1 mL/min, tR = 10.5 min. Figure 6. radio-HPLC spectra of [177Lu]Lu-AAZTA5-PSMA with linear gradient condition of 5–95% MeCN (+ 0.1% TFA)/95–5% Water (+ 0.1% TFA) in 10 min, 1 mL/min, tR = 10 min. Table 1. Stability values of 68Ga complexes of AAZTA5, AAZTA5-TOC and AAZTA5-PSMA-617. Table 2. Stability values of 44Sc and 177Lu complexes of AAZTA5, AAZTA5-TOC and AAZTA5-PSMA-617 [file 41181_2020_107_MOESM1_ESM.docx]

Supplementary Information:

PC3-negative cells:

**Figure 1** Internalization data of the ^44^Sc complexes of AAZTA^5^-PSMA-617, AAZTA^5^ and PSMA-617 in PC3 cells at 37 °C and 4 °C.

**Figure 2** Internalization data of the ^177^Lu complexes of AAZTA^5^-PSMA-617, AAZTA^5^ and PSMA-617 in PC3 cells at 37 °C and 4 °C.

Internalization with 4 °C values:

**Figure 3** Internalization data of the ^68^Ga, ^44^Sc and ^177^Lu complexes of AAZTA^5^-PSMA-617 and PSMA-617 in LNCaP cells at 37 °C and 4 °C.


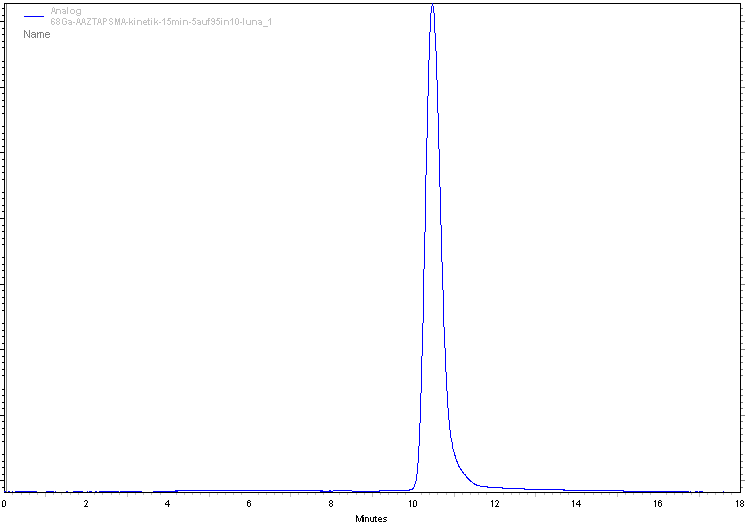


**Figure 4** radio-HPLC spectra of [^68^Ga]Ga-AAZTA^5^-PSMA with linear gradient condition of 5-95 % MeCN (+0.1 % TFA)/95-5 % Water (+0.1 % TFA) in 10 min, 1 mL/min, t_R_ = 10 min.


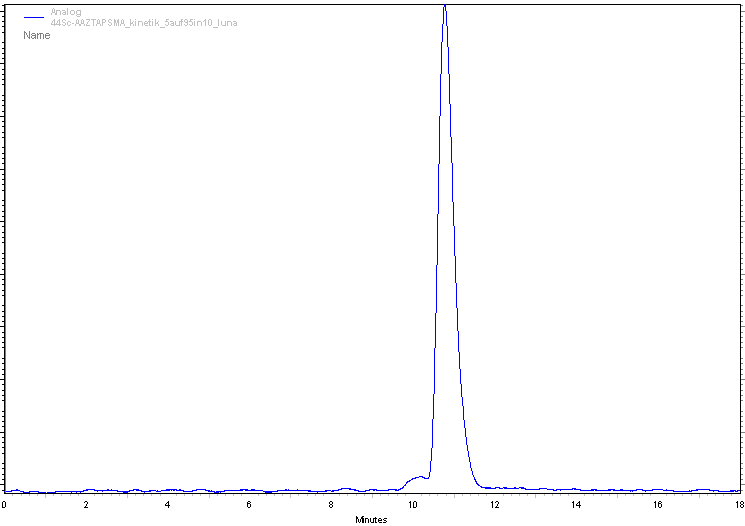


**Figure 5** radio-HPLC spectra of [^44^Sc]Sc-AAZTA^5^-PSMA with linear gradient condition of 5-95 % MeCN (+0.1 % TFA)/95-5 % Water (+0.1 % TFA) in 10 min, 1 mL/min, t_R_ = 10.5 min.


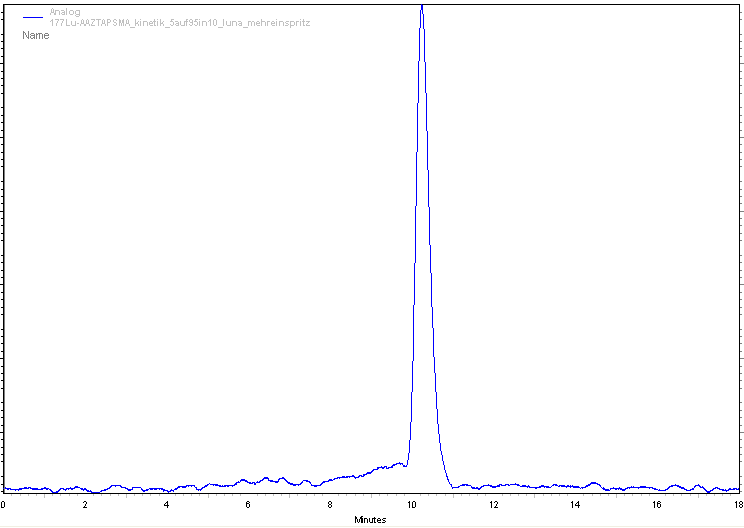


**Figure 6** radio-HPLC spectra of [^177^Lu]Lu-AAZTA^5^-PSMA with linear gradient condition of 5-95 % MeCN (+0.1 % TFA)/95-5 % Water (+0.1 % TFA) in 10 min, 1 mL/min, t_R_ = 10 min.

Full stability data:

**Table 1** Stability values of ^68^Ga complexes of AAZTA^5^, AAZTA^5^-TOC and AAZTA^5^-PSMA-617

|  | time | [^68^Ga]Ga-  AAZTA^5^ | [^68^Ga]Ga-AAZTA^5^-TOC | [^68^Ga]Ga-  AAZTA^5^-PSMA-617 |
| --- | --- | --- | --- | --- |
| HS | 0.5 h | 93.0 ± 0.8 | 98.1 ± 0.2 | 94.7 ± 0.3 |
|  | 1 h | 92.7 ± 1.3 | 96.9 ± 0.2 | 94.3 ± 0.4 |
|  | 2 h | 85.3 ± 3.4 | 95.0 ± 0.3 | 91.3 ± 0.7 |
| PBS | 0.5 h | 97.5 ± 1.0 | 99.1 ± 0.2 | 94.1 ± 0.2 |
|  | 1 h | 88.7 ± 0.5 | 99.2 ± 0.1 | 92.7 ± 0.5 |
|  | 2 h | 78.5 ± 4.5 | 99.3 ± 0.1 | 92.8 ± 0.6 |
| EDTA | 0.5 h | 93.0 ± 0.2 | 98.9 ± 0.4 | 96.8 ± 0.6 |
|  | 1 h | 50.3 ± 7.4 | 99.4 ± 0.2 | 96.0 ± 0,8. |
|  | 2 h | 49.5 ± 2.5 | 99.6 ± 0.1 | 92.3 ± 2.4 |
| DTPA | 0.5 h | 84.3 ± 0.9 | 97.8 ± 0.2 | 94.8 ± 0.9 |
|  | 1 h | 78.0 ± 1.4 | 99.4 ± 0.1 | 94.2 ± 0.8 |
|  | 2 h | 45.7 ± 1.3 | 99.6 ± 0.3 | 93.2 ± 3.6 |

**Table 2** Stability values of ^44^Sc and ^177^Lu complexes of AAZTA^5^, AAZTA^5^-TOC and AAZTA^5^-PSMA-617

|  | **^44^Sc** | | | | **^177^Lu** | | | |
| --- | --- | --- | --- | --- | --- | --- | --- | --- |
|  | time | AAZTA^5^ | AAZTA^5^-TOC | AAZTA^5^-PSMA | time | AAZTA^5^ | AAZTA^5^-TOC | AAZTA^5^-PSMA |
| HS | 1 h | 93.7 ± 0.9 | 95.1 ± 0.6 | 97.8 ± 1.3 | 1 h | 99.8 ± 0.1 | 92.5 ± 1.9 | 99.9 ± 0.1 |
|  | 4 h | 94.3 ± 1.7 | 94.3 ± 0.8 | 97.2 ± 0.3 | 2 h | 99.7 ± 0.1 | 92.3 ± 2.6 | 99.2 ± 0.4 |
|  | 8 h | 93.2 ± 1.1 | 95.0 ± 1.1 | 95.6 ± 0.8 | 24 h | 99.9 ± 0.1 | 91.5 ± 2.1 | 81.1 ± 2.1 |
|  | 24 h | 91.3 ± 1.8 | 93.8 ± 1.0 | 96.4 ± 4.0 | 7 d | 82.5 ± 3.2 | 86.3 ± 3.2 | - |
| PBS | 1 h | 95.3 ± 0.9 | 97.3 ± 0.5 | 96.2 ± 1.7 | 1 h | 97.8 ± 0.5 | 92.0 ± 1.4 | 99.4 ± 0.1 |
|  | 4 h | 92.7 ± 1.3 | 98.5 ± 0.9 | 96.4 ± 0.7 | 2 h | 98.6 ± 0.4 | 93.5 ± 1.3 | 99.3 ± 0.3 |
|  | 8 h | 95.9 ± 1.2 | 96.7 ± 1.4 | 94.6 ± 1.2 | 24 h | 99.1 ± 0.6 | 91.0 ± 2.2 | 90.1 ± 0.9 |
|  | 24 h | 96.0 ± 0.8 | 94.7 ± 1.6 | 86.9 ± 1.6 | 7 d | 96.0 ± 0.8 | 93.1 ± 1.8 | - |
| .EDTA | 1 h | 93.7 ± 1.3 | 96.0 ± 0.4 | 96.8 ± 1.8 | 1 h | 98.9 ± 0.2 | 95.7 ± 2.4 | 99.3 ± 0.2 |
|  | 4 h | 90.0 ± 3.2 | 97.6 ± 0.7 | 92.9 ± 2.0 | 2 h | 97.9 ± 0.3 | 95.1 ± 2.6 | 99.4 ± 0.1 |
|  | 8 h | 92.3 ± 2.1 | 96.9 ± 1.0 | 93.0 ± 0.7 | 24 h | 79.2 ± 2.9 | 86.8 ± 2.6 | 87.7 ± 1.8 |
|  | 24 h | 91.9 ± 1.8 | 95.4 ± 2.1 | 74.5 ± 4.8 | 7 d | 48.9 ± 1.5 | 77.5 ± 2.3 | - |
| DTPA | 1 h | 94.3 ± 0.9 | 98.7 ± 0.3 | 98.9 ± 0.4 | 1 h | 99.1 ± 0.2 | 98.9 ± 0.5 | 99.7 ± 0.1 |
|  | 4 h | 92.5 ± 0.5 | 98.9 ± 0.7 | 91.8 ± 2.7 | 2 h | 99.1 ± 0.1 | 96.2 ± 3.2 | 99.6 ± 0.1 |
|  | 8 h | 93.8 ± 1.2 | 96.9 ± 1.5 | 95.8 ± 0.3 | 24 h | 80.4 ± 5.4 | 82.8 ± 4.7 | 85.7 ± 1.5 |
|  | 24 h | 93.4 ± 1.1 | 92.6 ± 3.2 | 73.3 ± 2.3 | 7 d | 59.0 ± 14.3 | 72.9 ± 5.6 | - |

Data for AAZTA^5^, AAZTA^5^-TOC with ^68^Ga, ^44^Sc and ^177^Lu was taken from: Sinnes J., Nagel J., Rösch F., AAZTA^5^/AAZTA^5^-TOC: synthesis and radiochemical evaluation with ^68^Ga, ^44^Sc and ^177^Lu. EJNMMI Radiopharm. Chem. 4, 18 (2019).
